# Recent advances in plasmonic nanocavities for single-molecule spectroscopy

**DOI:** 10.1039/d0na00715c

**Published:** 2020-11-05

**Authors:** Nicolò Maccaferri, Grégory Barbillon, Alemayehu Nana Koya, Guowei Lu, Guillermo P. Acuna, Denis Garoli

**Affiliations:** Department of Physics and Materials Science, University of Luxembourg 162a avenue de la Faïencerie L-1511 Luxembourg Luxembourg; EPF-Ecole d'Ingénieurs 3 bis rue Lakanal 92330 Sceaux France; Istituto Italiano di Tecnologia Via Morego 30 16163 Genova Italy; State Key Laboratory for Mesoscopic Physics, Collaborative Innovation Center of Quantum Matter, Peking University Beijing 100871 China guillermo.acuna@unifr.ch; Département de Physique - Photonic Nanosystems, Université de Fribourg CH-1700 Fribourg Switzerland; Faculty of Science and Technology, Free University of Bozen-Bolzano Piazza università 1 39100 Bolzano Italy denis.garoli@unibz.it

## Abstract

Plasmonic nanocavities are able to engineer and confine electromagnetic fields to subwavelength volumes. In the past decade, they have enabled a large set of applications, in particular for sensing, optical trapping, and the investigation of physical and chemical phenomena at a few or single-molecule levels. This extreme sensitivity is possible thanks to the highly confined local field intensity enhancement, which depends on the geometry of plasmonic nanocavities. Indeed, suitably designed structures providing engineered local optical fields lead to enhanced optical sensing based on different phenomena such as surface enhanced Raman scattering, fluorescence, and Förster resonance energy transfer. In this mini-review, we illustrate the most recent results on plasmonic nanocavities, with specific emphasis on the detection of single molecules.

## Abstract 2

This mini-review reports the most recent results on plasmonic nanocavities applied to enhanced single-molecule detection and spectroscopy.

## Graphical abstract

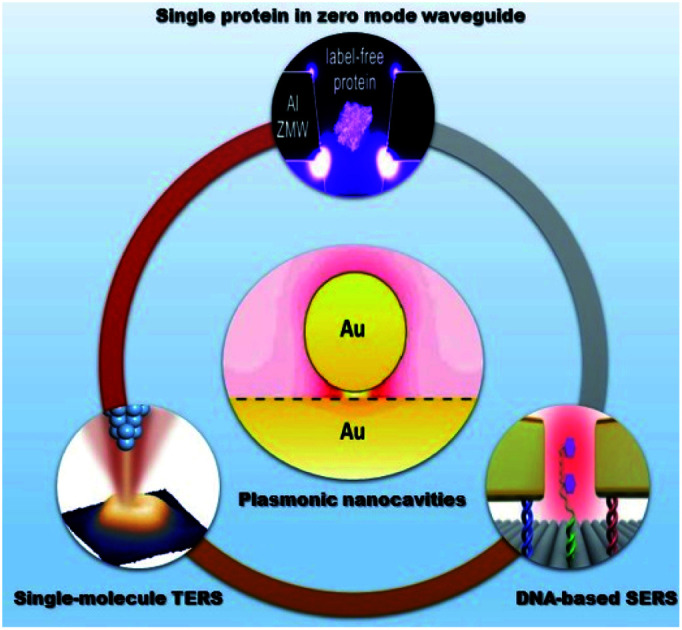

## Introduction

Single-molecule spectroscopy is a central topic in nanoscience, and tremendous applications have been developed so far, from sequencing and trapping^1^ to sub-nm control of quantum effects.^2–5^ In parallel, during the last decade, metallic plasmonic nanocavities were extensively investigated as transducers for enhanced sensing,^6,7^ optical trapping,^8^ single-molecule imaging^9^ and extreme nanophotonics.^10^ Plasmonic nanocavities enable the confinement of visible and near-infrared light to subwavelength volumes (typically a few tens of nm^3^) simultaneously, and the amplification of optical field intensity by several orders of magnitude.^3,11^ This local field intensity enhancement is possible thanks to the resonant excitation of surface plasmon polaritons generated from the coupling between the external electromagnetic (EM) radiation and the conduction electrons inside the metallic material. Thus, plasmonic nanocavities provide a powerful solution for reducing effective mode volumes and achieve, at the sub-nanometer scale, spatial control of the coupling with a single molecule in close proximity.^12,13^ Confining light to a cavity is then used to enhance the interaction between the optical field and low dimensional materials, including small molecules,^14^ 2D materials,^15^ quantum dots,^16^ nanoparticles^17,18^ or quantum emitters^5^ passing through or diffusing within the cavity.

A typical plasmonic nanocavity can be realized by coupling two nanostructures in a dimer-like fashion with a nanometer (or even sub-nanometer) gap. Alternatively, a dimer-like nanocavity can be achieved by placing a nanostructure above a metallic layer and separating the two building blocks by using a very thin (few nm) dielectric spacer. In this arrangement, also known as a nanoparticle-on-mirror (NPoM)^10,19^ cavity, the metallic nanostructure interacts with its image induced on the other side of the metallic layer. This image-charge configuration generates a plasmonic hotspot centered between the nanoparticle and the metallic substrate. NPoM nanocavities, which can support multiple resonances, exhibit deep sub-diffraction mode volumes below 10^−7^ (*λ*/*n*)^3^ (where *λ* is the incident radiation wavelength and *n* is the refractive index of the cavity).^20,21^ Finally, nanocavities can also be fabricated by engraving nanoholes of different shapes such as bow-tie, rectangular or circular, in thin metallic films.^22^ These geometries have been proved to be powerful platforms for many applications such as trapping and manipulation of nano-objects,^8,23^ bio-sensing,^24–26^ enhancement of the Raman signal of small molecules,^27^ and the realization of strong light–matter interactions.^2,28^ The present mini-review aims to collect the most recent results on various plasmonic nanocavity architectures, such as apertures in metallic films and zero-mode waveguides, pico-cavities with an atomic resolution, and nanocavities realized by using DNA-based nanofabrication techniques. In particular, we focus our attention on these architectures' extreme sensitivity capabilities to achieve single-molecule resolution.

### Apertures in metallic films: from zero-mode waveguides to plasmonic nanopores

**Fig. 1:**
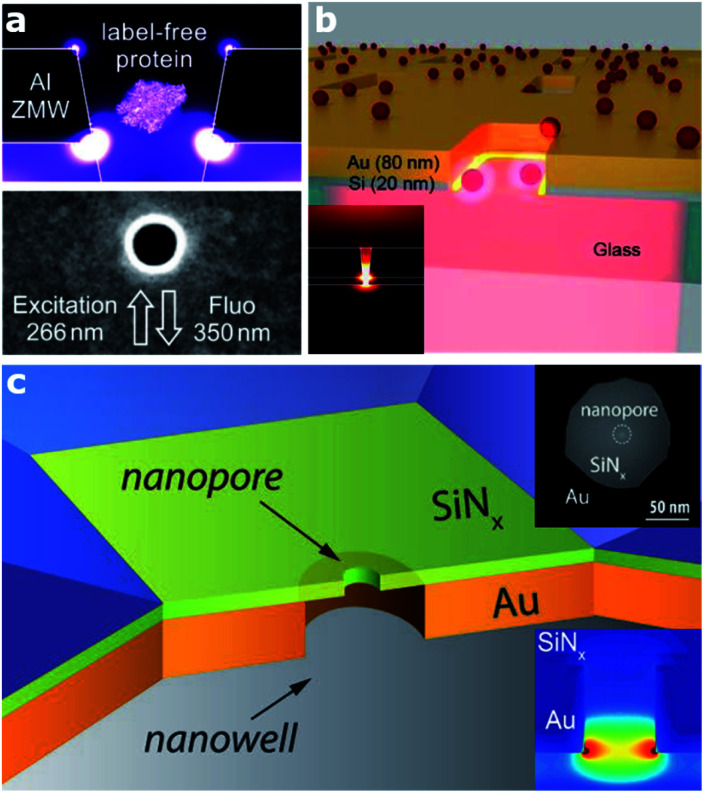
Various architectures of zero mode waveguide (ZMW) plasmonic nanoapertures. (a) Deep ultraviolet plasmonic enhancement of single protein autofluorescence in an Al ZMW. This figure has been reproduced from ref. 31 with permission from ACS Publications, copyright 2019. (b) A hybrid Au–Si zero mode waveguide for enhanced single molecule detection. This figure has been reproduced from ref. 30 with permission from the Royal Society of Chemistry, copyright 2019. (c) Enhanced single molecule fluorescence detection with a plasmonic nanowell–nanopore device architecture made of a nanowell fabricated in a gold film (orange) with a nanopore drilled in a freestanding Si_3_N_4_ membrane (light green). This figure has been reproduced from ref. 40 with permission from Wiley, copyright 2017.

In this section, we target a particular type of plasmonic nanocavity, which is often used to perform single-molecule detection by means of fluorescence. This architecture, a dubbed zero-mode waveguide (ZMW) since it operates at wavelengths longer than its cut-off wavelength, is realized by engraving an aperture, usually a square- or circular-like nanohole (typically with a lateral size of 50–100 nm), in a thin metallic film. This configuration allows guiding visible EM radiation into a volume smaller than its wavelength and confining it at the bottom of the aperture (Fig. 1). This extreme EM field confinement can reduce the effective detection volume down to 10^−21^ liter, allowing parallel and rapid sensing of molecules at concentrations in the micromolar range,^22,25,29–31^ since the excitation of molecules outside this detection volume is screened by the metallic film. Furthermore, besides affecting the excitation rate of the molecules inside the zeptolitre detection volume, ZMWs can also modify the fluorescence photokinetics decay rates,^32,33^ improving the net detected photon count rate per molecule.^22,34^ Although the most explored ZMW geometry is a circular hole prepared on a metallic film,^35^ recently several groups investigated alternative ZMW designs. In particular, rectangular ZMWs realized either on Al or Au–Si bilayers (Fig. 1(a) and (b), respectively), have been proved to yield significant enhancement both in terms of fluorescence signals and volume reduction.^22,29,30^

Alternatively, the use of apertures in metallic films and whose depth is partially engraved in a transparent substrate have been proved as a potential approach to increase the fluorescence of molecules.^34^ Current efforts in ZMW optimization are also devoted towards extending their working spectral range down to the UV, where several bio-molecules have intrinsic fluorescence, thus enabling label-free detection.^26,31^ Thanks to ZMW technology, a wide range of applications have been enabled, from DNA sequencing^36^ to enzymatic reactions.^25^ Moreover, the development of sensing architectures based on the ZMW concept paved the way for the realization of a new class of sensing platforms, so called solid-state nanopores,^37^ and consequently the subclass of plasmonic nanopores.^1^ Nanopore technology recently got massive attention, in particular for single molecule detection and sequencing.^38–40^ In the last few years, several groups investigated different configurations of plasmonic nanopores. A very smart as well as simple geometry has been proposed by Meller and co-workers, who realized a ZMW on a transparent thin Si_3_N_4_ membrane in a flow-through configuration by drilling a sub-10 nm hole in the membrane using a high-resolution transmission electron microscope^40^ (Fig. 1(c)). This platform enables enhanced single-molecule fluorescence detection and can be integrated with an electrical read-out of DNA translocation through the nanopore. Several optimizations or variations of this design have been reported. In terms of both local field confinement and enhancement, an outstanding example is represented by a bowtie antenna (dimer made of Au triangles) fabricated in close proximity to a solid-state nanopore.^38^

### Spectroscopic techniques used in plasmonic-based single-molecule sensing

Although fluorescence is the most used spectroscopic method in ZMW and nanopore-based single-molecule experiments, other phenomena have been investigated with very interesting outcomes. For instance, Förster resonance energy transfer (FRET) enhancement has been demonstrated in a ZMW.^25,26,41^ The exploitation of the FRET mechanism in plasmonic nanopores has been recently proposed as an efficient approach to multiplex maximum fluorescence wavelength channels by means of life-time/intensity multiplexing.^42,43^ The latter can find application in nanopore protein sequencing, where the high number of distinct amino acids to be discriminated (20) makes the fluorescence-based sequencing far more challenging. Moreover, plasmonic nanocavities have been demonstrated to enable forbidden dipole–dipole FRET exchanges.^24,44,45^

In the case of non-fluorescent molecules, surface-enhanced Raman scattering (SERS) spectroscopy performed on plasmonic nanocavities has been demonstrated to enable single-molecule sensitivity.^46–51^ The ability of SERS to detect these fingerprints with single-molecule sensitivity can be of paramount importance in plasmonic-based sequencing applications. In fact, by probing the SERS signals of four nucleotides and DNA oligonucleotides, the vibrational modes of single molecules very close (∼1 nm) to a metallic nanopore can be detected, owing to the enhanced electromagnetic fields.^52^ It is well-known that every molecule has its own Raman fingerprint, which is related to the building blocks of bio-molecules such as DNA, RNA, and proteins. In attempts to reach high and reproducible SERS signals, several types of plasmonic pores have been tested in recent years showing that the approach is potentially a winning strategy. As a significant example, recently van Dorpe and colleagues were able to detect DNA adsorbed inside a plasmonic nanoslit, reporting a spectroscopic library of nucleotides identified with single-molecule sensitivity.^52^

### From nano to picocavities for high-resolution single-molecule imaging and spectroscopy

**Fig. 2:**
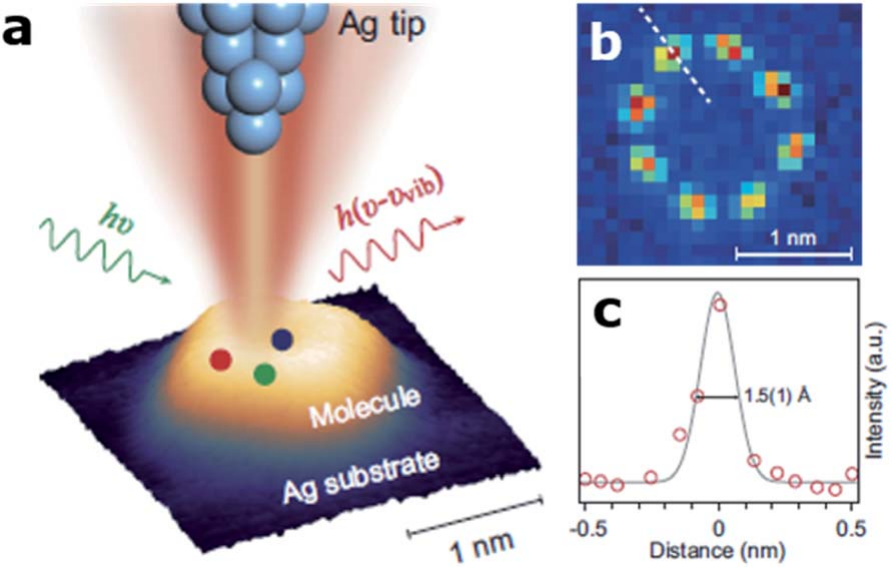
Ångström-resolved Raman images of vibrational modes for a single molecule by scanning Raman picoscopy (SRP). (a) The nanocavity defined by the silver tip and substrate generates a strong and highly confined plasmonic field, which is used for the excitation and emission enhancement of the Raman signals from a single molecule. (b) SRP image at 3072 cm^−1^ used for the estimation of spatial resolution. (c) Line profile of Raman signal intensities corresponding to the dashed line in (b), exhibiting a lateral spatial resolution down to 1.5(1) Å. This figure has been reproduced from ref. 55 with permission from Oxford Academic, copyright 2019.

Visualizing single molecules with chemical recognition represents a fundamental target in nano-biotechnology. Vibrational spectroscopy based on tip-enhanced Raman scattering (TERS) allows accessing the spectral signals of molecular species very efficiently *via* the strong localized plasmonic fields produced at the tip apex.^53^ In this context, recently Jaculbia and co-workers reported nanocavity-based TERS as a versatile tool for single molecule chemical analysis at the nanoscale.^47^ Similarly, nanocavities can be exploited to reconstruct single molecules' spatial locations within a plasmonic hotspot with an accuracy of 1 nm, thus enabling nanoscopy of their vibrational signatures.^54^ Recently, Hou and colleagues were able to image individual vibrational modes at the Ångström level for a single Mg-porphine molecule, revealing distinct characteristics of the vibrational modes in real space (Fig. 2).^55^ The same group also demonstrated spatially and spectrally resolved photoluminescence imaging of a single phthalocyanine molecule, as well as the local mapping of the molecular exciton energy and linewidth, coupled to nanocavity plasmons in a tunnelling junction with a spatial resolution down to ∼8 Å.^56^ Similarly, Lee *et al.* reported similar results using TERS at the precisely controllable junction of a cryogenic ultrahigh-vacuum scanning tunneling microscope, showing that Ångström-scale resolution is attained at subatomic separation between the tip atom and a molecule in the quantum tunneling regime of plasmons.^57^ They were able to record the vibrational spectra of a single molecule, obtain images of normal modes and analyse at the atomistic level the intramolecular charges and currents driven by vibrations.

**Fig. 3:**
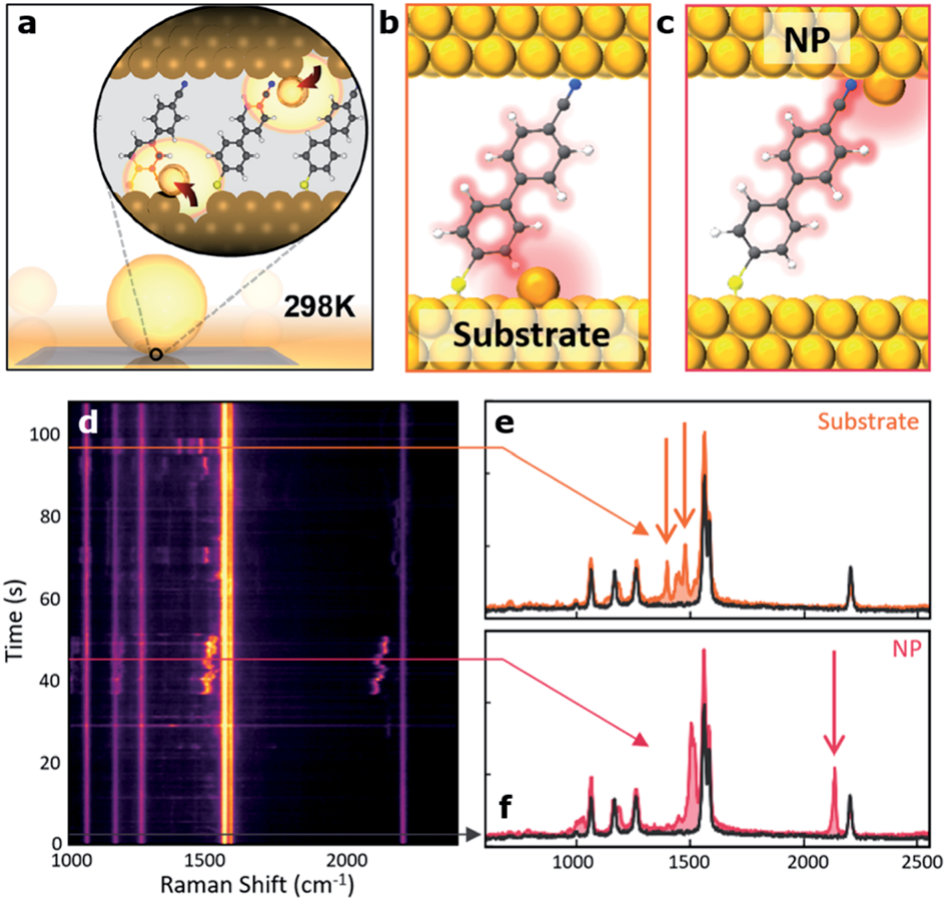
Room-temperature optical picocavities below 1 nm^3^ for accessing single-atom geometries. (a) Schematic of a nanoparticle-on-mirror (NPoM) geometry. The inset shows the formation of a picocavity by the movement of the surface atom to the adatom. (b, c) Schematic of picocavity adatoms on the Au substrate and nanoparticle facet, giving different interactions with 4-cyanobiphenyl-4-thiol (NC-BPT). (d) Consecutive SERS spectra showing transient peaks resulting from both forms of the picocavity. (e) SERS spectra for the picocavity adatom on the Au substrate and (f) picocavity adatom on the nanoparticle facet. This figure has been reproduced from ref. 60 with permission from ACS Publications, copyright 2018.

In this context, in pioneering work Baumberg and colleagues were able to place a self-assembled monolayer of biphenyl-4-thiol molecules sandwiched in a picocavity made of a gold nanoparticle on top of a gold film and able to localize light to volumes well below 1 nm^3^. This architecture was then used to experimentally record time-dependent Raman spectra from individual molecules at cryogenic temperature.^58^ They reported extreme optical confinement yielding a 100-fold enhancement, thus enabling optomechanical coupling between the cavity field and the vibrations of individual molecular bonds. In the same year they also showed that by scaling the cavity volume to less than 40 nm^3^, they could achieve room temperature strong coupling at the single-molecule level.^59^ Recently, the same group reported large-scale room-temperature single-molecule detection by using nanocavities to retrieve either the enhanced Raman scattering of the molecules (Fig. 3)^60^ or their fluorescence emission.^4^

Finally, it is worth mentioning here that narrow fingerprint Raman peaks are promising for biomedical analysis. Raman sensing based on a flow-through scheme is desirable for many practical applications, including lab-on-chip diagnostics. Recently, Huang *et al.* introduced a new scheme to achieve on demand control and delivery of single plasmonic nanoparticles, which can be functionalized with Raman tags. Using an analogous strategy and exploiting the enhanced optical field in a picocavity formed by translocating a gold nanoparticle coupled to a plasmonic nanohole, they were able to discriminate the SERS signal of single DNA bases in single oligonucleotides by an electro-plasmonic trapping mechanism.^27^ They also used this approach to detect single amino acid residues in polypeptides.^61^

### Nanocavities realized by using DNA-based nanotechnology

**Fig. 4:**
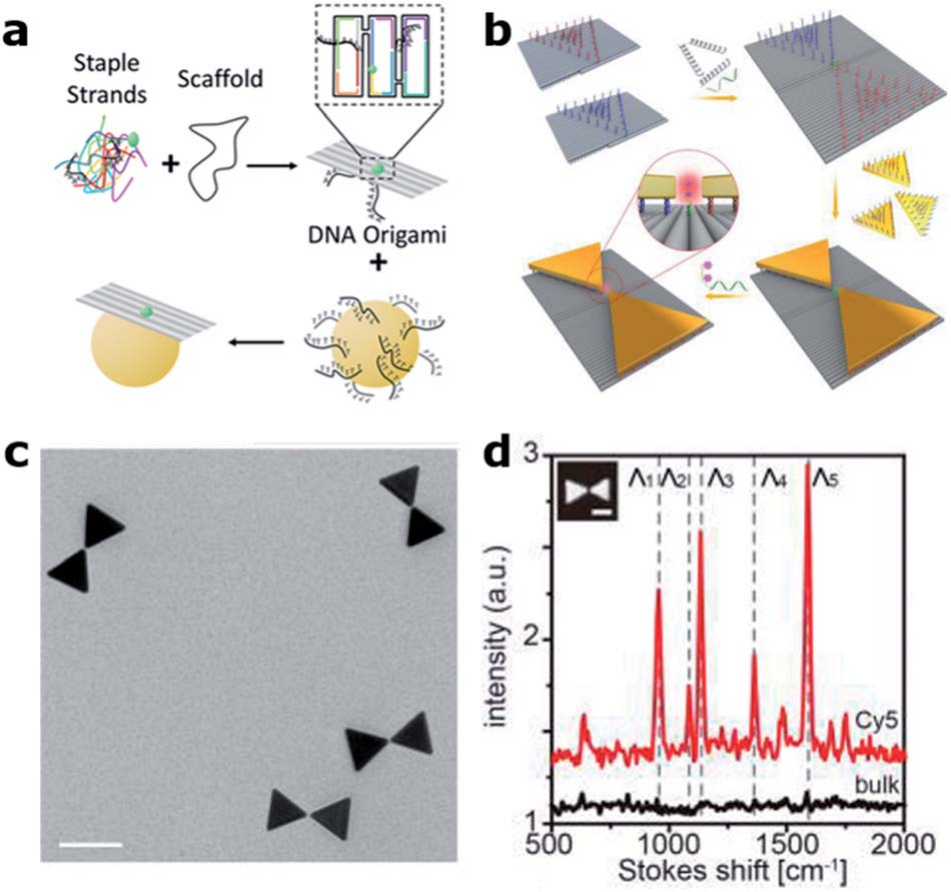
DNA origami based nanocavities. (a) Basic principle: a “long” ss-DNA sequence (Scaffold) is folded with the help of hundreds of “short” ss-DNA sequences into the predesigned shape. MNPs, previously functionalized with DNA, can be incorporated through DNA hybridization. (b) Bowtie antennas self-assembled onto DNA origami structures fabricated based on two rectangular DNA origami structures. A single Cy5 Raman active molecule is incorporated at the bowtie hotspot. (c) Corresponding TEM image (scale bar 50 nm) and (d) Raman spectra of an individual bowtie with a single Cy5 (red) and a bulk solution of Cy5 (black). (b)–(d) have been reproduced from ref. 76 with permission from Wiley, copyright 2018.

Over the last decade, the DNA origami technique has been consolidated into the state-of-the-art approach for the self-assembly of nanophotonic structures.^62,63^ DNA origami is fabricated in a bottom-up manner by folding a “long” single-stranded DNA (ss-DNA) sequence (termed “scaffold”, ∼8000 bases long) into a predesigned shape with the help of approximately 200 “short” ss-DNA sequences (termed “staples”, ∼40 bases long and complementary to the scaffold sequence – see Fig. 4(a)). These structures can be used as breadboards where different species, including single-photon emitters such as organic fluorophore molecules and quantum dots, together with colloidal metallic nanoparticles (MNPs) of different shapes, materials and sizes, can be incorporated with nanometric accuracy and stoichiometric control^64^ to form nanocavities.

Compared to conventional “top-down” nanofabrication techniques, *i.e.*, electron or ion beam lithography, the DNA origami technique has three main advantages. First, it is a bottom-up self-assembly process in which billions of structures can be fabricated in a parallel fashion without the need of costly equipment. Second, it employs colloidal MNPs, which are less prone to surface defects and can be fabricated with higher uniformity than evaporated metallic structures leading to improved reproducibility and performance.^65^ Finally, with this technique, a single-photon emitter can be routinely placed in the hotspots of MNPs with nanometer precision, a key factor in controlling the coupling between single molecules and nanocavities.^66,67^ These advantages were initially exploited to revisit experiments on fluorescence-enhanced spectroscopy^68,69^ and SERS^70–72^ using dimer nanocavities made of Au or Ag, achieving enhancement values outperforming in some cases those obtained by using nanocavities fabricated with more complex top-down lithographic techniques.^73^ While most DNA origami based dimer nanocavities are based on spherical MNPs, anisotropic geometries such as gold nanorods were also demonstrated.^74,75^ One step further was recently taken by Ding's group by positioning and orienting triangular gold nanoplates onto two rectangular DNA origami structures in order to fabricate bowtie antennas^76^ (see Fig. 4(b) and (c)). The advancement introduced by the DNA origami fabrication technique is reflected by smaller and more homogenous gaps between the triangular plates reaching 5 ± 1 nm, which represents an extremely challenging gap to fabricate with lithographic techniques. Moreover, smaller gaps translate into a 200-fold higher electric field enhancement. However, the main advantage of this approach is that single Cy3 and Cy5 Raman active molecules could be placed at the hotspot of the bowtie antenna in order to demonstrate single-molecule SERS (Fig. 4(d)).

**Fig. 5:**
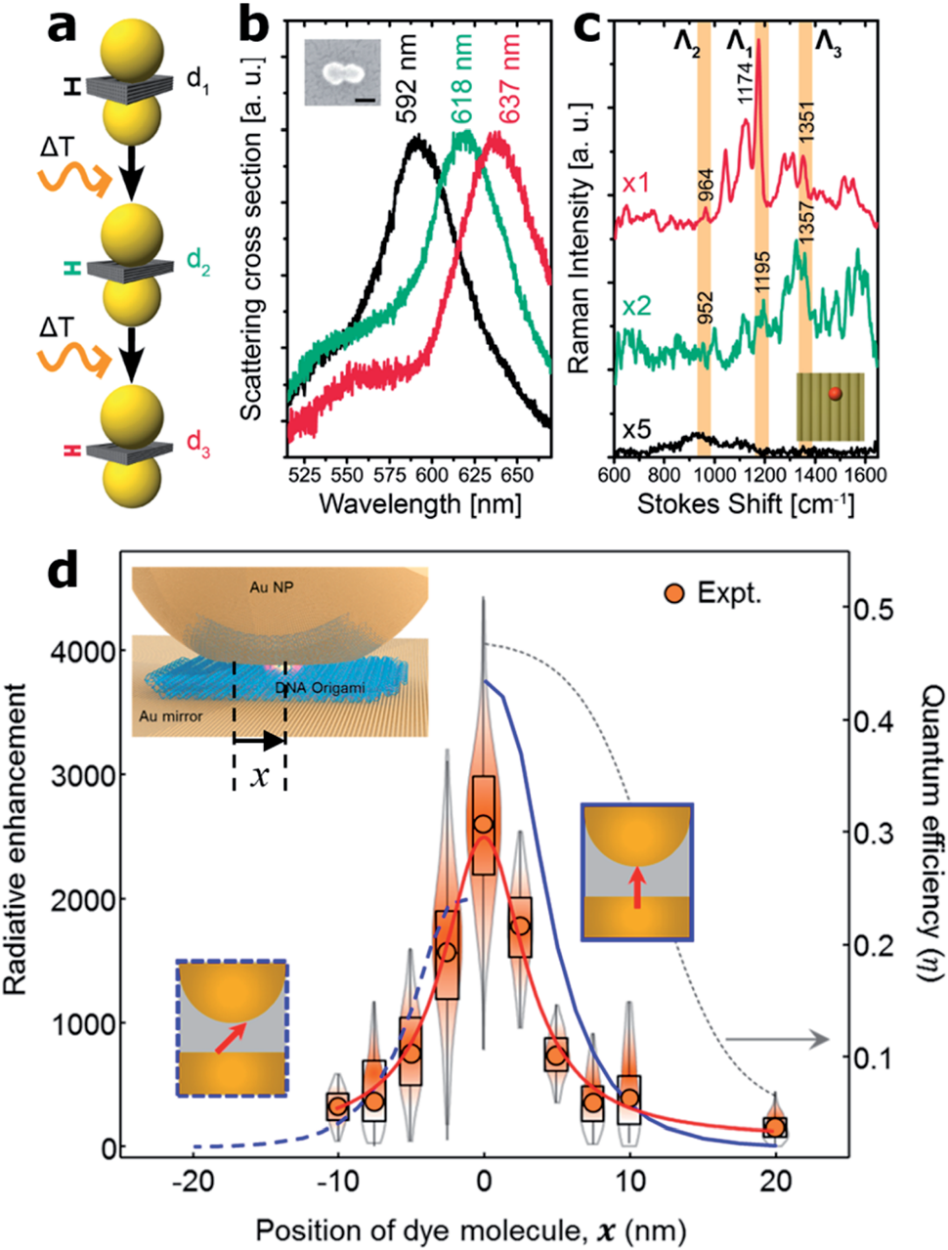
Gap control and NPoM nanocavities. (a) Sketch of the Au MNP dimer bound to a rectangular DNA origami structure for thermal control of the nanocavity gap. (b) Scattering cross-section as the dimer gap is reduced by increasing the temperature. (c) Scattering spectra of an individual dimer structure modified with a single Cy3.5 molecule placed at the hot spot before (black) and after a first (green) and a second (red) round of a 10 s laser excitation (612 nm, ∼60 kW cm^−2^). This figure has been reproduced from ref. 49 with permission from ACS Publications, copyright 2016. (d) Fluorescence measurements on different samples in which the single molecule was laterally displaced. Values were normalized to the emission on bare glass. Inset: sketch of the fabrication approach of the NPoM using a DNA origami as a spacer. A single molecule can be placed at the NPoM hotspot with nanometer precision. This figure has been reproduced from ref. 77 with permission from ACS Publications, copyright 2018.

Another improvement recently enabled by the DNA origami technique is the possibility to tune the gap of dimer nanocavities. The groups led by Liedl and Lohmüller showed that the distance between two Au 40 nm MNPs self-assembled onto a DNA origami structure can be adjusted by increasing the cavity temperature, see Fig. 5(a). An increase of approximately 200 °C leads to a shrinking of the DNA molecule and thus to a reduction of the gap size from 2.5 to 1.4 nm.^49^ The gap thermal shrinking was monitored by studying the redshift in the scattering cross-section of the dimer nanocavity (Fig. 5(b)) and by SERS measurements of a single Cy3.5 molecule placed at the hotspot.

An alternative approach for the fabrication of nanocavities, using the NPoM geometry, was proposed by Chikkaraddy and co-workers.^77^ A rectangular DNA origami structure was used as a spacer in order to self-assemble a single spherical Au MNP onto an Au layer forming an NPoM nanocavity (Fig. 5(c)). This approach enabled the subsequent incorporation of single molecules at a well-defined position within the NPoM cavity gap in contrast to initial studies where neither the position nor the stoichiometry of fluorescent molecules could be controlled.^78^ This level of position control was exploited to map the hotspot of the NPoM cavity by placing single Cy5 molecules at different positions within the 5 nm gap between an 80 nm gold MNP and a thick gold layer using a rectangular DNA origami (Fig. 5(d)). By performing fluorescence measurements at each position, the spatial profile of the local density of optical states was estimated with a resolution of approximately 2 nm.^77^

## Conclusions

In summary, since their development, plasmonic nanocavities have gained growing interest due to their unique capabilities to confine and concentrate the EM field in the nanometer and sub-nanometer range. This effect has been exploited to apply spectroscopic techniques (*e.g.*, SERS and fluorescence) to detect single molecules with a strong impact on biomedical applications such as real-time DNA sequencing. ZMWs and plasmonic nanopores still represent the most valuable platforms for single diffusing/translocating molecule detection at high concentrations in the micromolar range. They can be used not only in single-molecule spectroscopy, but also integrated with additional functionalities such as optical trapping and thermo-electro-phoretic effects. Moreover, advanced nanofabrication approaches enable the preparation of hybrid devices that integrate both solid-state and biological elements (such as organic coatings or functional proteins).^79,80^ Future development in nanocavities engineering might include the exploration of high-index dielectric materials for the fabrication of dimers,^81,82^ and their integration with MNPs.^83^ Other directions might include the combination of dimers and NPoM structures, so called dimers-on-film, in order to obtain narrow resonances through the superposition of bright and dark modes.^84,85^ DNA nanotechnology might also play a fundamental role in optimizing nanocavity hotspots. This paves the way for the combination of nanocavities with, for example, bio-assays for DNA sensing and diagnostics.^86^ Finally, we also envision a synergistic combination of the DNA origami technique with nanoapertures^87,88^ in order to control and address their occupation.

## Conflicts of interest

There are no conflicts to declare.

## Supplementary Material
